# Population Structure and Implications on the Genetic Architecture of HIV-1 Phenotypes Within Southern Africa

**DOI:** 10.3389/fgene.2019.00905

**Published:** 2019-09-27

**Authors:** Prisca K. Thami, Emile R. Chimusa

**Affiliations:** ^1^Division of Human Genetics, Department of Pathology, University of Cape Town, Cape Town, South Africa; ^2^Research Laboratory, Botswana Harvard AIDS Institute Partnership, Gaborone, Botswana

**Keywords:** population structure, diversity, genome-wide association studies (GWAS), host genetics, Southern Africa, HIV-1

## Abstract

The interesting history of Southern Africa has put the region in the spotlight for population medical genetics. Major events including the Bantu expansion and European colonialism have imprinted unique genetic signatures within autochthonous populations of Southern Africa, this resulting in differential allele frequencies across the region. This genetic structure has potential implications on susceptibility and resistance to infectious diseases such as human immunodeficiency virus (HIV) infection. Southern Africa is the region affected worst by HIV. Here, we discuss advances made in genome-wide association studies (GWAS) of HIV-1 in the past 12 years and dissect population diversity within Southern Africa. Our findings accentuate that a plethora of factors such as migration, language and culture, admixture, and natural selection have profiled the genetics of the people of Southern Africa. Genetic structure has been observed among the Khoe-San, among Bantu speakers, and between the Khoe-San, Coloureds, and Bantu speakers. Moreover, Southern African populations have complex admixture scenarios. Few GWAS of HIV-1 have been conducted in Southern Africa, with only one of these identifying two novel variants (*HCG22*rs2535307 and *CCNG1*kgp22385164) significantly associated with HIV-1 acquisition and progression. High genetic diversity, multi-wave genetic mixture and low linkage disequilibrium of Southern African populations constitute a challenge in identifying genetic variants with modest risk or protective effect against HIV-1. We therefore posit that it is compelling to assess genome-wide contribution of ancestry to HIV-1 infection. We further suggest robust methods that can pin-point population-specific variants that may contribute to the control of HIV-1 in Southern Africa.

## Introduction

Southern Africa extends across a 2.7 million km^2^ land in the southernmost part of Africa and is the home to about 66 million people (Worldometers, 2019). The region comprises 10 mainland countries: Angola, Botswana, Lesotho, Malawi, Mozambique, Namibia, South Africa, eSwatini, Zambia, and Zimbabwe (Marks, 2014). The region carries the highest burden of human immunodeficiency virus/acquired immune deficiency syndrome (HIV/AIDS). Around 37 million people live with HIV globally, and over half of these live in Southern and eastern Africa (UNAIDS, 2017). Despite high exposure to HIV, some people stay uninfected (Fowke et al., 1996), while those infected exhibit heterogeneous clinical outcomes of HIV infection and to an extent also differential antiretroviral drug metabolism (Carr et al., 2017). This heterogeneity is partly due to underlying diversity in host genetics (Telenti and Goldstein, 2006).

**Figure 1:**
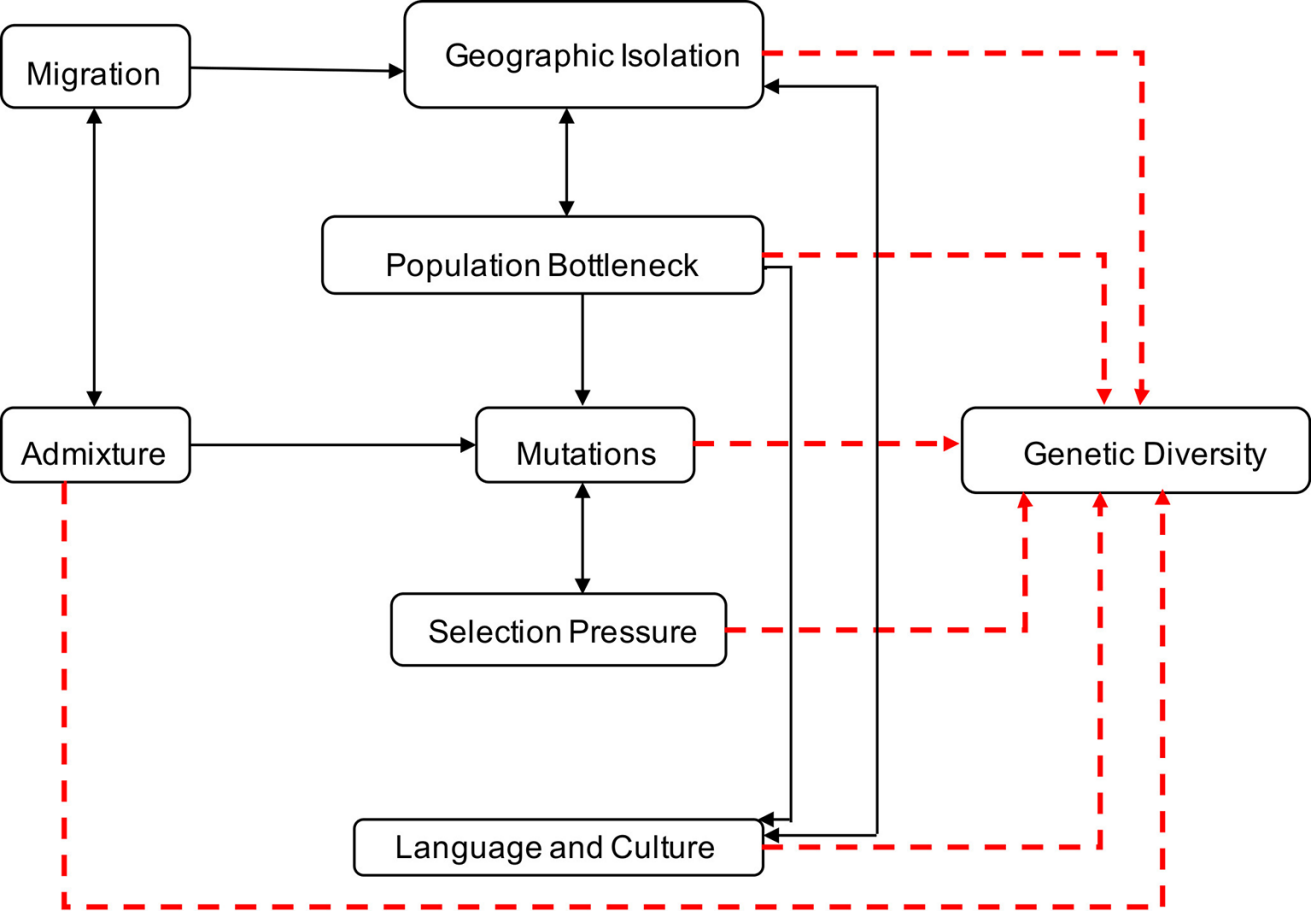
Factors contributing to human genetic diversity within Southern Africa. Although it is complex to represent the interplay between geographic isolation, migration, population bottleneck, admixture, language, culture, and natural selection on the genetic structure of Southern Africa, this figure provides a basic illustration of some of the factors that shaped the genetics of the people of Southern Africa. Geographic distribution and migration into Southern Africa have influenced population admixture and geographic isolation, yielding to population bottleneck, mutation, and selection, that shaped the genetics, culture, and language diversity in Southern Africa.

Genetic diversity within and between human populations can be inferred from the observed distributions of genetic allele frequencies in populations, a concept also known as population (genetic) structure (Wright, 1950; Underhill and Kivisild, 2007). The classic measure of genetic population structure is Wright’s fixation index, F_ST_, a type of ANOVA statistic (Tishkoff and Williams, 2002; Jakobsson et al., 2013). The value ranges from zero to one, with F_ST_ scores close to one indicating a high degree of population divergence (i.e., most genetic variation between populations), and F_ST_ scores close to zero indicating strong population similarity and gene flow (i.e., most genetic variation within populations) (Tishkoff and Williams, 2002). Geographic isolation, migration, population bottleneck, admixture, language and culture, and natural selection have played significant roles in contributing to variations in allele frequencies and the genetic landscape of Southern Africa (Campbell and Tishkoff, 2008; Campbell and Tishkoff, 2010; Botigue et al., 2013; Breton et al., 2014; Gurdasani et al., 2014; Li et al., 2014; Macholdt et al., 2014; Chimusa et al., 2015) as illustrated in **Figure 1**.

Genome-wide association studies (GWAS) are approaches of scanning the genome to identify *in silico* variants that confer susceptibility or resistance to a particular disease through statistical association models (Hirschhorn and Daly, 2005; Hutcheson et al., 2008). Unlike candidate gene-based methods that require *a priori* knowledge of suspected genes, GWAS have the potential to discover novel genomic loci (Telenti and Goldstein, 2006). Although GWAS are powerful techniques, the variants discovered through these methods (Purcell et al., 2007; Kang et al., 2010; Yang et al., 2011; Wen et al., 2018) have not accounted for all of the variability in viral load (Fellay et al., 2007; Fellay et al., 2009; Pereyra et al., 2010). The overall heritability of set point viral load in populations of European ancestry measured through GWAS was estimated to be 24.6%. Common variants contributed largely to this estimate of heritability (McLaren et al., 2015; Tough and McLaren, 2019). Like other complex traits, this highlights the importance of solving the missing heritability of HIV-1 infection phenotypes which might be uncovered by discovering factors such as rare variants, structural variants, and gene-gene and gene-environment interactions responsible for inter-host variability of viral load (Verma and Ritchie, 2018).

Confounders such as population structure can affect GWAS results. These have to be controlled to avoid spurious results (Hirschhorn and Daly, 2005; Price et al., 2006; Tishkoff et al., 2009; McLaren and Carrington, 2015). Moreover, characterizing genetic structure is crucial for reconstruction of human population history (Tishkoff et al., 2009). In general, African populations have the highest genetic variation and lower linkage disequilibrium (LD) among loci (Campbell and Tishkoff, 2008; The 1000 Genomes Project Consortium, 2010; Choudhury et al., 2018); therefore, not all tag-single nucleotide polymorphism (SNPs) selected from other populations can be used as proxies in African populations. Risk alleles can be structured in populations due to multiple demographic factors and genetic ancestry contributions (Botigue et al., 2013; Gurdasani et al., 2014; Chimusa et al., 2015; Skoglund et al., 2017). The people of Southern Africa are culturally, linguistically, and genetically diverse; the region has been underrepresented in previous genetic diversity studies (Awany et al., 2018; Choudhury et al., 2017; Sirugo et al., 2019).

Most GWAS were performed in non-African populations (Awany et al., 2018; Sirugo et al., 2019) in which HIV-1B is the prevalent subtype. It is possible that the genetics underlying the control of HIV-1 in Southern African is different from these other populations. Considering these genetic differences between African and other populations, and due to the enormous burden of HIV within Southern Africa, it is imperative to dissect human genetic diversity and investigate the role of genetic landscape on HIV acquisition and progression within the region. Deducing a comprehensive architecture of HIV host genetics in Southern Africa will assist in the development of population-specific interventions against HIV. Hence, this review aims to present a comprehensive discussion of the advances made in the GWAS of HIV-1 and document common variants within Southern Africa associated with HIV-1 infection.

We used PubMed search engine to retrieve HIV-1 GWAS studies which have been published in the past 12 years (2007–2019); species was restricted to the human species. The specific search terms were the following:((“genome”[MeSH Terms] OR “genome”[All Fields]) AND wide[All Fields] AND (“association”[MeSH Terms] OR “association”[All Fields]) AND (“hiv-1”[MeSH Terms] OR “hiv-1”[All Fields])) AND (“2007/01/01”[PDat]: “2019/04/30”[PDat] AND “humans”[MeSH Terms]). Ninety-eight items were retrieved; articles relevant to Southern Africa were used in the review. Cited studies which were not in the search results were directly searched for. To review population structure and admixture in Southern Africa, a relaxed search of the terms (population structure and Southern Africa; human genetic diversity and Southern Africa; admixture and Southern Africa) was performed in PubMed, and relevant articles were selected for this review. SNP annotations were confirmed on dbSNP (Sherry et al., 2001). A map of migration routes (refer to the *Migration Into Southern Africa* section) was created using maps package in R and edited using MacOS Preview software. We conclude with a discussion of research areas where further work on GWAS of HIV-1 is needed.

## Migration Into Southern Africa

Demic diffusion has been shown to have a paramount role in genetic drift and gene flow, the two most important events that can trigger population structure. The major expansions of humans date as far back as 100,000 years ago (the Out of Africa (OOA) model) (Campbell and Tishkoff, 2008). One of the major human expansions that occurred in relatively recent years is the Bantu expansion that happened 3,000–5000 years ago. The Bantu dispersed from western Africa, in the region known as the proto-Bantu homeland in eastern Nigeria and western Cameroon (Cavalli-Sforza et al., 1993; Soodyall, 2006; de Filippo et al., 2010; Li et al., 2014).

Migration routes of the Bantu populations have been well described (Li et al., 2014). According to archeological, linguistics, and genetics evidence, two main theories of the split of Bantu speakers from their western African homeland have been postulated: a) migration of the eastern Bantu speakers directly from western Africa to eastern Africa, and thereafter the ancestors of Nguni and Sotho-Tswana speakers moved between 1,200 and 1,000 years into Southern Africa through Great Zimbabwe (Soodyall, 2006; Li et al., 2014) and b) migration of western Bantu speakers through southern Cameroon directly to Southern Africa. A hypothesis supported by Bayesian tree methods stated that the eastern and western Bantu speakers might have split later after passing through the central African rainforest (Li et al., 2014).

**Figure 2:**
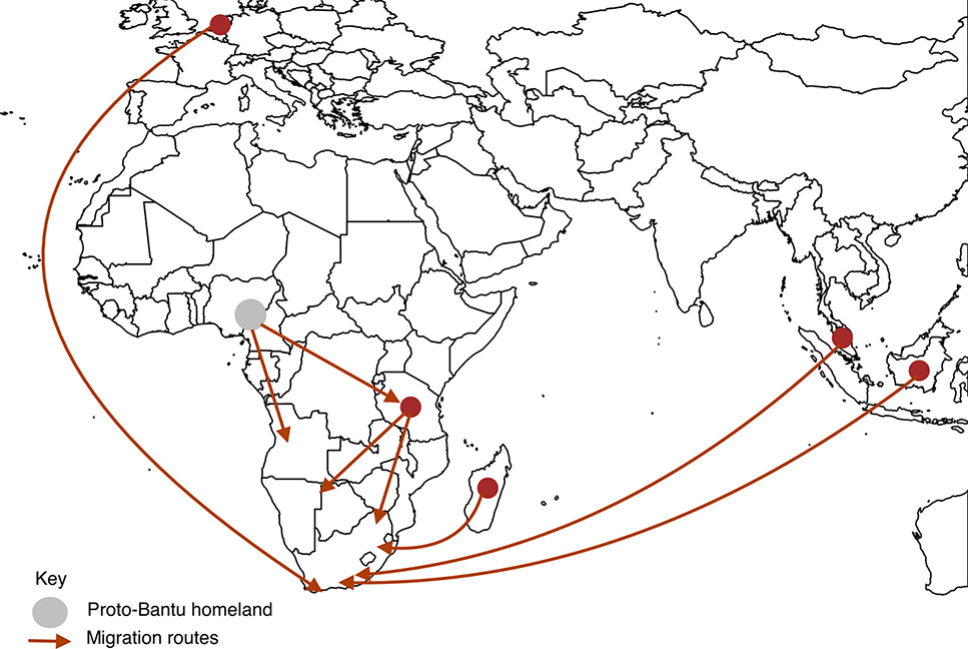
Migration routes into Southern Africa. We present here the main routes of pre-historic migration of the ancestors of the Southern African inhabitants. The circles represent places of origin, the gray circle being the proto-Bantu land and dark red circles being other places of origin. The arrows represent movement of people from places of origin into Southern Africa.

In more recent history, Southern Africa has seen an influx of migrants from other continents such as Europe and Asia. By 1750 the Dutch and Portuguese had colonized Southern Africa on the southwest and east coasts, respectively (Hall, 1993). These colonial powers brought slaves and exiles from Indonesia, Malaysia, east coast of Africa, Madagascar and India, to provide labor in wine and wheat farms in Cape Town, the then Cape of Good Hope (Hall, 1993; de Wit et al., 2010). Intermarriages and liaison between these populations resulted in progeny who are now termed “Coloureds” (de Wit et al., 2010). The major migration routes into Southern Africa are depicted in **Figure 2**.

## Language and Cultural Diversity in Southern Africa

The inhabitants of Southern Africa could be distinctly classified according to ways of subsistence and food production: hunters and gatherers (and fishers) and the agro-pastoralists (Phillipson, 1977). It has been suggested that the distribution of languages in Southern Africa is an effect of demic diffusion rather than language diffusion alone (Huffman, 1970; Li et al., 2014). Southern Africa was previously occupied by hunter-gatherers who had their own unique culture. Food production was a major drive in human expansions as evidenced in the spread of farming from the cradle in western Africa (Neumann et al., 2012; Russell et al., 2014). Farming was able to support higher population densities (population explosion) than hunting and gathering, which gave farmers an edge over the indigenous inhabitants. As such the culture and languages of hunter-gatherers have been replaced by that of early farmers (Diamond and Bellwood, 2003).

In general, African populations can be classified into four main ethnolinguistic groups: Niger-Kordofanian, Afroasiatic, Nilo-Saharan, and Khoisan (Tishkoff et al., 2009). Over 200 million sub-Saharan inhabitants speak a collective of related languages known as Bantu languages. Bantu speakers are a sub-group of the Niger-Kordofanian ethnolinguistic group (Heine and Nurse, 2000; Reed and Tishkoff, 2006; Campbell and Tishkoff, 2010; Li et al., 2014; Veeramah and Hammer, 2014). Majority of Southern African inhabitants are Bantu speakers and Khoisan-speakers. Over 50 Bantu languages are spoken in Southern Africa Eberhard et al. (2019), these include but are not limited to Tswana, Nama, Xhosa, Zulu, Ndebele, Swati, Sotho, Shona, Bemba, and Makua (Heine and Nurse, 2000; Soodyall, 2006).

Khoisan language families include Khoe, !Xun (Ju), Ta’a (including !Xo), !Wi, and Southwestern or Cape /Xam. The Khoe and the San intermixed resulting in linguistic affinities and diffusion of culture between the two groups; this led to them collectively being referred to as the Khoe-San (Schlebusch, 2010; Schlebusch et al., 2016). The present-day Khoe-San inhabit the central Kalahari Desert and Okavango swamps of Botswana, much of Namibia, Angola, Zimbabwe, Cape Province, and some parts of Lesotho. It is believed that the Khoe-San occupied most of Southern Africa before the arrival of farmers who displaced the Khoe-San to the desert (Barnard, 1992; Li et al., 2014). The Khoe-San are the indigenous inhabitants of Southern Africa. Analysis of genome-wide data and the lactase (LCT) region has shown that the Khoe originated from the assimilation of the San into eastern African Bantu-speaking populations (Schlebusch et al., 2012; Breton et al., 2014; Sadr, 2015; Schlebusch et al., 2016).

Using autosomal SNPs, the Pygmy (a Central Africa population), the Hadza, and Sandawe clustered together with Southern African Khoe-Sans. This suggested that the Pygmy, Southern African Khoe-San, Hadza, and Sandawe populations are a remnant of a proto-Khoe-San-Pygmy population (Tishkoff et al., 2009). Although there have been postulations of the San also originating in East Africa (Soodyall, 2006; Campbell and Tishkoff, 2008; Gonder et al., 2007; Barbieri et al., 2013a; Morris et al., 2014), the origin of the San in Southern Africa cannot be ruled out. In fact, Nurse et al. (1985) suggests that by the 17th century the San lived in most parts of Southern Africa. Before the arrival of Bantu speakers, the Southern San inhabited the foothills of the Drakensberg mountains, the northern, eastern, and western Cape provinces of South Africa, and the southmost parts of Botswana and Namibia [Supplementary information (Schlebusch et al., 2016)]. The rock paintings in Tsodilo Hills in northwestern Botswana show that the Northern San had been living around Tsodilo Hills by the first millennium AD before the arrival of the Khoe and the Bantu speakers.

The Southern African Khoe-San underwent a population bottleneck due to barriers such culture, language, and geographic isolation (i.e., the Kalahari Desert, which could not allow their interaction and contact with other populations). The Khoe-San also underwent genocide at the hands of Bantu speakers and European settlers. This stemmed from conflicts over land which the colonists had invaded while the Khoe-San also hunted the colonists’ cattle, as they used to hunt antelopes and elands (Adhikari, 2010; Schlebusch et al., 2016). The population decline was also due to the Khoe-San succumbing to diseases such as smallpox which were introduced by the settlers (Chimusa et al., 2015). It is speculated that smallpox might have been introduced into the Cape through passengers from India and then spread to European settlers who might have passed it to the Khoe-San. Since the Khoe-San had less resistance to the disease, this drastically reduced their population numbers. The acquisition of Khoe-San women as wives by the European settlers due to the small number of European women also diminished the gene pool of the Khoe-San, this further exacerbating the bottleneck of the Khoe-San population (Nurse et al., 1985).

Bantu speakers were agro-pastoralists, while the Khoe were pastoralists and the San were hunter-gatherers (Breton et al., 2014). There has been some cultural (Breton et al., 2014) and language diffusion within Southern Africa. Through interaction, the San acquired the pastoralism lifestyle from the Bantu and Khoe populations (Breton et al., 2014). Although the click consonants have been connected to the Khoe-San, these have been borrowed into some of the Bantu groups such as the Zulu and Xhosa populations (Barbieri et al., 2013a; Marks et al., 2015). Interactions and trade among Southern African populations has resulted in cultural diffusion which has driven intermixing of the different populations; this has in turn shaped the genetics of Southern African populations.

## Population Genetic Diversity in Southern Africa

The gene pool of Southern Africa is a mosaic of alleles from multiple ancestries (Montinaro et al., 2017). Past historical events such as geographical isolation, colonialism, and the Bantu expansion have helped to shape the genetic landscape of Southern Africa (Choudhury et al., 2017). When determining variation between the Khoe and San, using two genetic marker systems, mitochondrial DNA (mtDNA) and Y-chromosome, Soodyall and colleagues could not unambiguously distinguish the Khoe from the San (Soodyall et al., 2008). According to the authors, the Khoe and the San share a common gene pool, which suggests that they branched off from a common ancestry.

Conversely, genome-wide studies analyzing millions of SNPs revealed that the Khoe and the San are a genetically diverse group (Schlebusch et al., 2012). Schlebusch et al. (2012) analyzed 2.3 million SNPs of autosomal chromosomes from 220 individuals representing 11 populations from Southern Africa. These populations were Ju/’hoansi, !Xun, /Gui and //Gana, Karretjie, ‡Khomani, Nama, Khwe, “Coloured” (Colesberg), “Coloured” (Wellington), Herero, and Bantu speakers (South Africa). In their report, there was a distinct stratification in the Khoe-San group; there was a separation between the Ju speakers (!Xun and Ju/’hoansi) who are the San, and the Nama representing Khoe speakers. This is consistent with the findings of a study by Uren et al. (2016) who found fine-scale structure between the ‡Khomani and Nama of South Africa.

The Khoe-San are genetically distinct from the Bantu populations (**Table 1**). In the Schlebusch et al. (2012) study, a variant associated with “fast-twitching” muscles and elite athletic performance had greater frequencies (>90%) in the Khoe-San groups than in other Southern African populations. Moreover, a strong signal of selection was found around the human leukocyte antigen (HLA) complex in several genes that are known to protect against infectious diseases in the ‡Khomani and Karretjie, this effect probably owing to early and extensive contact with European colonists and novel infectious diseases such as smallpox (Schlebusch et al., 2012). Likewise, Tau and colleagues also reported significant genetic differences between the Khoe-San and Bantu-speaking groups through analysis of short tandem repeats (STRs) (Tau et al., 2017).

**Table 1:** Genome-wide population diversity studies of Southern African populations.

Population	Country	Genotyping method	Marker	Major findings	Reference
Khoe-San and Bantu speakers	Namibia, South Africa	Microarray (genome-wide)	Autosomal SNPs and CNVs	Khoe-San and Bantu speakers are genetically different.	(Jakobsson et al., 2008; Li et al., 2008)
Bantu speakers, SAC and Khoe-San	South Africa	Panel sequencing	Autosomal SNPs, indels, and microsatellites	Khoe-San, SAC and Bantu speakers are genetically divergent. SAC show the highest level of intercontinental admixture.	(Tishkoff et al., 2009)
Khoe-San and Bantu speakers	Botswana, Namibia, South Africa	Microarray (genome-wide)	Autosomal SNPs	Structure observed among the Khoisan, and between the Khoisan, SAC, and Bantu speakers.	(Pickrell et al., 2012; Schlebusch et al., 2012)
Khoe-San	South Africa	Microarray (genome-wide)	Autosomal SNPs	Precolonial Eurasian admixture observed in Khoe-San populations.	(Pickrell et al., 2014)
Khoe-San	Angola, Botswana, South Africa	Microarray (genome-wide)	Autosomal SNPs, mtDNA, and Y-chromosome	Khoe-San show high genetic differentiation and have sex-biased Bantu speakers and European admixture.	(Henn et al., 2011; Montinaro et al., 2017; Uren et al., 2016; Choudhury et al., 2017; Oliveira et al., 2019)
Bantu speakers and SAC	South Africa	Microarray (genome-wide) and WGS	Autosomal SNPs	Multiway admixture including Khoe-San ancestry observed in the SAC population. Differential Khoe-San admixture detected in Bantu speakers.	(de Wit et al., 2010; Chimusa et al., 2013a, Gurdasani et al., 2014; Chimusa et al., 2015; Uren et al., 2016; Choudhury et al., 2017; Chimusa et al., 2018)
Bantu speakers	South Africa	Microarray (genome-wide)	Autosomal SNPs	Weak clustering of southeastern Bantu speakers possibly due to admixture with Khoe-San.	(May et al., 2013)
Khoe-San and Bantu speakers	South Africa, Namibia, Malawi	Microarray (genome-wide)	Autosomal SNPs	Admixture in Malawian population supports the late split of the Eastern Bantu speakers. Eurasian admixture detected in South African Khoe-San, dating back to European colonial period settlement in Southern Africa.	(Busby et al., 2016)
Bantu speakers and Khoe-San	Botswana	Microarray (whole exome) and WES	Autosomal SNPs	Within population structure of Botswana was not observed. Genetic differentiation was observed between Sotho, Zulu (of South Africa), and the Botswana population.	(Retshabile et al., 2018)

Indels, insertions/deletions; SAC, The “Coloured” of South Africa; SNP, single nucleotide polymorphism; CNV, copy number variation; mtDNA, mitochondrial DNA; WES, whole exome sequencing; WGS, whole genome sequencing. Details on specific groups within Khoe-San and Bantu speakers can be obtained from original publications.

In another study, SNPs associated with lactase persistence (LP) from eight Khoe-San and seven Bantu-speaking groups from Botswana, Namibia, and Zambia were characterized (Macholdt et al., 2014). LP is a distinctive trait of pastoralism. LP alleles were first identified in eastern Africa (Tishkoff et al., 2007) where the alleles have faced a strong positive selection. In the study conducted by Macholdt et al. (2014), -14010*C was the most frequent SNP with a collective higher prevalence in pastoralists (20.2%) than in foragers (6.7%) or agriculturalists (1.3%). Within the pastoralists group, -14010*C was observed at the highest frequency of 36% in the Nama population. This further shows the implication of migration and gene flow from the east of Africa into Southern Africa through Khoe speakers.

Y-STR analysis of Southern African populations revealed regional variation between the populations of Botswana, Zimbabwe, Namibia, and South Africa (Tau et al., 2015). Whole genome sequences of Southern African populations also revealed genetic variation among the Bantu of South Africa and among Khoe-San groups. Principal component analysis and structure analysis revealed significant genetic differentiation (p < 10^-6^) between the Xhosa and Sotho groups (Choudhury et al., 2017). F_ST_ analysis revealed genomic regions with high divergence between the two groups (Average F_ST_ ≥ 0.3) (Choudhury et al., 2017; Supplementary Table 11). There have been reports of a weak clustering of Bantu-speaking populations that might be underpinned by admixture (May et al., 2013; Retshabile et al., 2018). Southern (and eastern) African populations have greater genetic diversity than other populations closer to the western African origin. This might be due to admixture that arose from the mixing of eastern and Southern African Bantu populations with indigenous populations of the regions (Schlebusch et al., 2012; Chan et al., 2015). Admixture between genetically isolated and differentiated populations can result in a mosaic of population-specific chromosomal blocks (Daya et al., 2014; Sanderson et al., 2015; Chimusa et al., 2018).

## Patterns of Admixture in Southern Africa

Bantu groups reached Southern Africa 1,200–2,000 years ago (Ramsay et al., 1996; Soodyall, 2006; Barbieri et al., 2014) and reached the southeastern Cape 1,300 years ago (Breton et al., 2014). Arriving in Southern Africa, Bantu groups did not replace indigenous populations entirely; instead, there have been assimilations of the indigenous populations into the Bantu groups through intermixing and intermarriages especially between migrant men and Khoe-San women (Nurse et al., 1985; Diamond and Bellwood, 2003; Barbieri et al., 2014; Li et al., 2014).

L0d and L0k mtDNA haplogroups have been previously shown to be localized in the Khoe-San (Behar et al., 2008; Barbieri et al., 2013b; Schlebusch et al., 2013). Barbieri et al. (2014) examined mtDNA haplogroups L0d and L0k to measure admixture between the Khoe-San and Bantu speakers. The presence of these mtDNA lineages in Bantu-speaking populations may suggest very ancient admixture with the Khoe-San (Barbieri et al., 2013b). The authors showed that these haplogroups were shared with the Khoe-San, with a group such as the Kgalagadi of southern Botswana harboring 53% frequency of the haplogroups (Barbieri et al., 2014). A considerable amount of admixture was also observed between the Bantu speakers of South Africa (Zulu and Xhosa populations) through elucidation of Y-chromosome haplogroups (Naidoo et al., 2010). Consistent with this finding, Gurdasani et al. (2014) found hunter-gatherers admixture across African populations, the greatest level of admixture being in Zulu and Xhosa populations.

Y-chromosome and mtDNA analyses of Southern African populations suggest patterns of sex-biased migration and admixture (Underhill and Kivisild, 2007). The Bantu expansion was possibly a male-dominated event which led to Khoe-San females being assimilated into Bantu-speaking populations (Bajic et al., 2018). Henn et al. found that approximately 35% of paternal lineages in the Khoe-San group were either of Bantu or European origin (Henn et al., 2011). This sex-biased interaction between migrant men and Khoe-San females has led to asymmetrical patterns of admixture in the region (**Table 1**). A common pattern is the flow of Y-chromosome from migrant men into Khoe-San populations or the flow of mtDNA from Khoe-San women into migrant populations (Choudhury et al., 2017; Bajic et al., 2018; Oliveira et al., 2019).

Precolonial admixture of indigenous Southern African populations with populations of west Eurasian ancestry has also been documented (Pickrell et al., 2014). Due to the age of this admixture and suggestive linguistic, archeological, and genetic evidences of migration into Southern Africa from eastern Africa, Pickrell et al. (2014) concluded that the precolonial west Eurasian ancestry in Southern Africa is due to the indirect gene flow through eastern Africa. Signatures of admixture in Khoe-San groups have also been observed. In their study, Pickrell and colleagues detected ∼6% non-Khoe-San ancestry in the Ju’|hoan North (Pickrell et al., 2012). Moreover, a genetic component common in Southeastern Bantu speakers was observed in most Khoe-San groups from Botswana, Lesotho, and South Africa (Montinaro et al., 2017).

The “Coloured” of South Africa (SAC) are another popular, well-studied population in Southern Africa (**Table 1**). The SAC show the highest level of intercontinental admixture, with high levels of Southern African Khoisan, Niger-Khodofanian, Indian, and European ancestries and low levels of Asian and Cushitic ancestries (Tishkoff et al., 2009). Consistent to this insight, a multi-faceted admixture scenario (five-way admixture) has been documented in a study that sought to develop a method (PROXYANC) that could detect the best proxy ancestry in admixture in the SAC population (Chimusa et al., 2013a). The results of this study suggested the admixture to be a combination of Europeans (16%), Xhosa (33%), Gujarati (12%), Chinese (7%), and ‡Khomani (31%) ancestries.

Recent direct admixture involving Eurasian groups has been detected using GLOBETROTTER, an R program, (Hellenthal et al., 2014) in the ‡Khomani and Karretjie of South Africa, dating back to five generations (220 years ago) which aligns with the period of European settlers’ arrival in Southern Africa (Busby et al., 2016). Congruent with this, in a recent study Chimusa et al. (2018) dated admixture in the SAC population to approximately 4 ± 1 generations ago, which likely reflects the wave of admixture that occurred during the era of colonialism in Southern Africa.

Admixture mapping is increasingly becoming an indispensable tool for predicting diseases in admixed populations. Admixture mapping is a method of measuring the contribution of ancestry to a phenotype (Smith et al., 2004; Chimusa et al., 2015; Chi et al., 2019). In Southern Africa, a GWAS of tuberculosis (TB) in the SAC population revealed a link between San ancestry and susceptibility to *Mycobacterium tuberculosis* (Chimusa et al., 2013b; Daya et al., 2014).

## History of HIV in Southern Africa

AIDS was first recognized as a new disease in 1981 when multiple young homosexual men in Los Angeles and New York presented with opportunistic infections and a rare form of Kaposi’s Sarcoma (Greene, 2007; Sharp and Hahn, 2011). HIV was later confirmed as the causative agent of AIDS (Gallo et al., 1984). There are two types of HIV: HIV-1 and HIV-2. HIV-1 is classified into three groups; Main group (M), Outlier group (O), and non-M-non-O group (N). Due to high mutation and recombination rates of HIV-1, the pandemic group, M, is further divided into genetic subtypes A–D, F–H, J–K, and intersubtype recombinants, as well as circulating recombinant forms (CRFs) and unique recombinant forms (URFs). Over half of global HIV infections are caused by HIV-1. HIV-1 group M has a worldwide distribution; subtype A is more prevalent in eastern Europe, subtype B in North America, South America, and western Europe, and subtype C in Southern Africa and India. HIV-2, which is more prevalent in western Africa, is divided into subtypes A–H and is less prevalent and less pathogenic than HIV-1 (Hemelaar et al., 2011; Eberle and Gurtler, 2012; Wilkinson et al., 2015).

**Figure 3:**
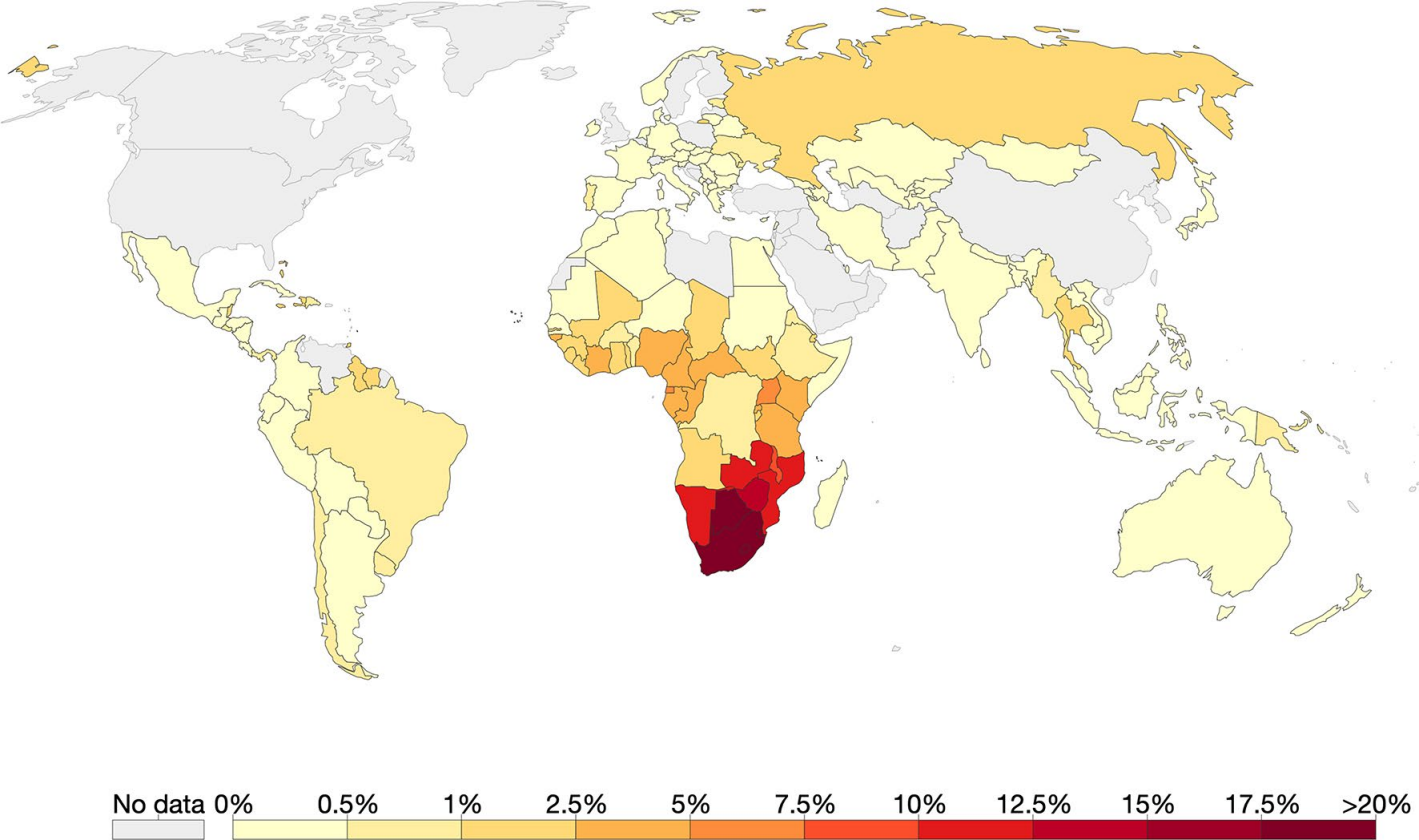
The global prevalence of HIV as of 2017. Of all the countries that had data, Southern Africa had the highest HIV prevalence. Source: Our World in Data; data provided by UNAIDS.

Southern Africa has the highest HIV prevalence globally with countries such as eSwatini leading with 27.4% [**Figure 3**, (Taha, 2011; Vermund et al., 2015)]. The predominant subtype in the region is HIV-1C (Wilkinson et al., 2015). Using coalescence and molecular clock methods, HIV-1C was traced to the late 1930s in the Democratic Republic of Congo (Wilkinson et al., 2015). This strain is believed to have later spread to Southern and eastern Africa (Hemelaar et al., 2011; Faria et al., 2014; Wilkinson et al., 2015). Although the first samples of HIV-1 were sampled in South Africa in 1985, the origin of HIV-1C epidemic in Southern Africa was placed around 1960 [95% highest posterior density (HPD) 1956–1964] (Wilkinson et al., 2015).

The first anti-HIV drug, azidothymidine (AZT) also known as zidovudine, was approved in 1987. This was an anti-cancer drug that was also found to inhibit reverse transcriptase (Greene, 2007). AZT and subsequent monotherapies were short-lived due to limitations including low genetic barrier to resistance and toxicity. This led to the introduction of combination therapy of multiple antiretrovirals [cART or highly active antiretroviral therapy (HAART)] with better tolerability and potency (Arts and Hazuda, 2012; Vella et al., 2012). In the early 1990s, there was high morbidity and mortality as a result of HIV in most Southern African countries (Kagaayi and Serwadda, 2016). The region has since seen a steep decline of 30% in HIV-related mortality between 2010 and 2017 due to rapid scale-up of antiretroviral therapy (UNAIDS, 2018). However, these drugs only suppress HIV to undetectable levels; they do not eliminate HIV from cellular reservoirs (McLaren and Carrington, 2015). While current therapeutic measures are geared towards elimination of HIV in cellular reservoirs and the use of broadly neutralizing antibodies against HIV, effective vaccination against HIV remains a hurdle due to ineffective immune responses against HIV and high variability of HIV sequence (Halper-Stromberg and Nussenzweig, 2016; Siliciano and Siliciano, 2016; Hsu and O’Connell, 2017).

Southern African countries concomitantly bear the brunt of both HIV and TB (Corbett et al., 2003; John et al., 2007; Abdool Karim et al., 2009; Dokubo et al., 2014; Tafuma et al., 2014). In 2017 an estimate of 10 million people developed active TB, and 72% of these were HIV-infected people living in Africa (WHO, 2018). HIV is the greatest predictor of development of active TB; susceptibility to active TB development increases 20-fold in people infected with HIV (Pawlowski et al., 2012). HIV/TB coinfection is the leading cause of mortality in Southern Africa even in people receiving antitubercular and antiretroviral therapy (Bisson et al., 2015; Campa et al., 2017). In a HIV/TB coinfection scenario, TB has a negative impact on immune response to HIV, thus exacerbating progression to AIDS (Bruchfeld et al., 2015). Another complication associated with HIV infection is the manifestation of adverse restoration of immune responses, known as immune reconstitution inflammatory syndrome (IRIS), which occurs during the initial months of antiretroviral treatment (Lawn et al., 2005). Although IRIS unmasks a spectrum of pre-existing opportunistic infections, it is commonly associated with mycobacterial infections (Lawn et al., 2005; Meintjes et al., 2008). TB-IRIS is a major health challenge in the developing world (Bruchfeld et al., 2015; Walker et al., 2018).

## Pre-GWAS of HIV-1: Candidate AIDS Restriction Genes

One of the striking characteristics of HIV infection is the highly heterogeneous viral set point across infected individuals. Set point is the viral load of an individual that fluctuates around a steady point during the asymptomatic phase of HIV infection (Fraser et al., 2007). The difference in viral set point between individuals can be as high as 1,000-fold (Mellors et al., 1996; Fellay et al., 2007; Fraser et al., 2007). Some of this variability is underpinned by host genetic factors. Following the discovery that HIV infected cells by binding to CD4 cells and CCR5, the first AIDS restriction gene (ARG) to be discovered was *CCR5*-Δ32 (Dean et al., 1996). Subsequently, a number of SNPs associated with HIV-1 were identified by candidate gene analysis in other ARGs (O’Brien and Nelson, 2004; Hutcheson et al., 2008; Winkler, 2008). These ARGs include those encoding HLA class I antigen presentation molecules (Carrington et al., 1999; Gao et al., 2001; Carrington and O’Brien, 2003; Welzel et al., 2007); chemokines and chemokine receptor genes involved in HIV-1 cell entry such as CCR2, CXCR6, and CCL5 (RANTES) (Smith et al., 1997; An et al., 2002; Duggal et al., 2003); natural killer cell immunoglobin-like receptors (KIR) genes (Martin et al., 2002); cytokines (Shin et al., 2000); and the intrinsic viral restriction factors TRIM5 (Javanbakht et al., 2006) and APOBEC3G (An et al., 2004). A detailed discussion of these SNPs and harboring genes can be found in these reviews (O’Brien and Nelson, 2004; Hutcheson et al., 2008).

Candidate gene methods have been pivotal in revealing genes that may be used as targets for drug development (van Manen et al., 2011). For instance, *CCR5* variants have enabled the development of HIV-1 entry inhibitor drugs (Troyer et al., 2011). Maraviroc, which is a CCR5 antagonist, is currently the only chemokine receptor in clinical use (Henrich and Kuritzkes, 2013). Through the identification of *CCR5*-Δ32, to date two people have maintained complete HIV-1 remission after a *CCR5*-Δ32 stem transplant (Hutter et al., 2009; UNAIDS, 2019). The ARGs identified by candidate gene analysis explained about 10% of the variability in HIV-1 infection (O’Brien and Nelson, 2004; Hutcheson et al., 2008). HapMap annotation of about 3 million human SNPs has facilitated the development of high-density arrays with 500,000 to 1,000,000 variants that could be utilized in the screening of variants associated with any disease (The International HapMap Consortium, 2003; Hutcheson et al., 2008; Winkler, 2008); this set the stage for HIV-1 GWAS.

## A Global Perspective of GWAS of HIV-1

GWAS are useful tools for identifying genetic contributions towards predisposition to infection, progression to disease, and differential drug metabolism (Adebamowo et al., 2017). GWAS offer an unbiased opportunity to screen the entire genome and pinpoint variants implicated in a given phenotype of interest. Here and in the subsequent section, we present an account of GWAS of HIV-1 as illustrated in **Table 2**.

**Table 2:** GWAS significant genes associated with HIV-1 acquisition, viral load set point, and progression.

Known gene	Description	Potential protein function	Effect	Population	Reference
*HCP5*	HLA complex P5	Related in sequence to human endogenous retroviruses and possibly interacts directly with HIV.	Minor alleles of SNPs rs2395029*, rs2255221, and rs2523608** were associated with low viral loads and delayed progression	Europeans, African Americans, and Africans	(Fellay et al., 2007; Dalmasso et al., 2008; Fellay et al., 2009; Limou et al., 2009; Pereyra et al., 2010; Le Clerc et al., 2011)
*HLA-B*	MHC class I B	Plays a critical role in the immune system; peptide presentation *via* endoplasmic reticulum (ER) pathway.	SNPs found in the gene were associated with low viral loads and delayed progression.	Europeans, Chinese, African Americans, and Africans	(Fellay et al., 2009; Herbeck et al., 2010; Pelak et al., 2010; Pereyra et al., 2010)
*HLA-C*	MHC class I C	Plays a critical role in the immune system; peptide presentation *via* endoplasmic reticulum (ER) pathway.	Minor allele of rs9264942 was associated with low viral loads and delayed progression.	Europeans	(Fellay et al., 2007; Fellay et al., 2009; Pereyra et al., 2010)
*ZNRD1*	Zinc ribbon domain containing 1	Plays a role in regulation of cell proliferation.	SNPs identified in this gene were associated with low viral loads and delayed progression.	Europeans	(Fellay et al., 2007, Fellay et al., 2009)
*TNXB*	Tenascin XB	Plays a role in matrix maturation during wound healing.	SNPs identified in this gene were associated with low viral loads.	Europeans	(Dalmasso et al., 2008)
*TNF*	Tumor necrosis factor	Cytokine. Involved in the regulation of biological processes including cell proliferation, differentiation, apoptosis, lipid metabolism, and coagulation.	Minor allele of SNP rs3093662 was associated with low viral loads.	Europeans	(Dalmasso et al., 2008)
*SDC2*	Syndecan 2	Mediates cell binding, cell signaling, and cytoskeletal organization. Syndecan receptors are required for internalization of the HIV-1 tat protein.	Minor allele of SNP rs2575735 was associated with reduction in HIV reservoir.	Europeans	(Dalmasso et al., 2008)
*DDX40YPEL2*	DEAH-Box Helicase 40 Yippee Like 2	NR	Minor allele of intergenic rs6503919 was associated with low viral reservoir.	Europeans	(Dalmasso et al., 2008)
*TRIM10*	Tripartite motif containing 10	Plays a role in terminal differentiation of erythroid cells.	SNP rs9468692 was associated with low viral loads.	Europeans	(Fellay et al., 2009)
*NOTCH4*	Notch receptor 4	Regulates interactions between physically adjacent cells.	Minor allele of SNP rs8192591 was associated with low viral loads and delayed progression.	Europeans	(Fellay et al., 2009; Le Clerc et al., 2011)
*RNF39*	Ring finger protein 39	Plays a role in early phase of synaptic plasticity.	SNPs found in the gene were associated with low viral loads and delayed progression.	Europeans	(Fellay et al., 2009; Limou et al., 2009)
*C6orf48*	Small nucleolar RNA host gene 32	NR	Minor allele of SNP rs9368699 was associated with low viral loads and delayed progression.	Europeans	(Limou et al., 2009; Le Clerc et al., 2011)
*PSORS1C1*	Psoriasis susceptibility 1 candidate 1	Confers susceptibility to psoriasis and systemic sclerosis.	SNPs found in the gene were associated with low viral loads.	Europeans	(Limou et al., 2009)
*MICB*	MHC class I polypeptide-related sequence B	Involved in immune response. Activates natural killer cells, CD8 alphabeta T cells, and gammadelta T cells.	SNPs found in the gene were associated with low viral loads.	Europeans	(Limou et al., 2009; Herbeck et al., 2010)
*SOX5*	Sex determining region Y -box 5	Regulation of embryonic development and determination of the cell fate.	Minor allele of SNP rs1522232 was associated with delayed progression.	Europeans	(Le Clerc et al., 2009)
*RXRG*	Retinoid X receptor gamma	Mediates the antiproliferative effects of retinoic acid.	Minor allele of SNP rs10800098 was associated with rapid progression.	Europeans	(Le Clerc et al., 2009)
*TGFBRAP1*	Transforming growth factor beta receptor associated protein 1	Acts as a chaperone in signaling downstream of TGF-beta.	Minor allele of SNP rs1020064 was associated with delayed progression.	Europeans	(Le Clerc et al., 2009)
*PROX1*	Prospero homeobox 1	Plays a role in development.	A haplotype of rs17762192, rs17762150, and rs1367951 was associated with delayed progression.	Europeans	(Herbeck et al., 2010)
*MICA*	MHC class I polypeptide-related sequence A	A ligand for the NKG2-D type II receptor that acts as a stress-induced antigen that is broadly recognized by intestinal epithelial gamma delta T cells.	Minor allele of SNP rs4418214 was associated with low viral loads.	Europeans	(Pereyra et al., 2010)
*PSORS1C3*	Psoriasis susceptibility 1 candidate 3	NR	Minor allele of SNP rs3131018 was associated with low viral loads.	Europeans	(Pereyra et al., 2010)
*AL671883.2 DHFRP2*	Pseudogenes	NR	Minor allele of intergenic SNP rs2523590 was associated with low viral loads.	Europeans, African Americans, Hispanics	(Pereyra et al., 2010)
*HCG22*	HLA complex group 22	NR	Minor allele of SNP rs9262632 was associated with low viral loads in African Americans. Minor allele of SNP rs2535307 was associated with rapid progression and increased susceptibility.	African Americans and Southern Africans	(Pereyra et al., 2010; Xie et al., 2017)
*RICH2*	Rho GTPase activating protein 44	Involved in GTPase activation activity and phospholipid binding.	Major allele of SNP rs2072255 was associated with rapid progression.	Europeans	(Le Clerc et al., 2011)
*PARD3B*	Par-3 family cell polarity regulator beta	May play a role in asymmetrical cell division and cell polarization processes.	SNP rs11884476 was associated with delayed progression.	Europeans	(Troyer et al., 2011)
*CCR5*	C-C motif chemokine receptor 5	Acts as a co-receptor for macrophage-tropic viruses to enter host cells.	CCR5-Δ32 was associated with resistance to HIV-1 infection.	Europeans	(McLaren et al., 2013)
*CCNG1*	Cyclin G1	May play a role in cell proliferation and regulation	SNP kgp22385164 was associated with rapid progression.	Southern Africans	(Xie et al., 2017)

Potential function of genes according to Pubmed and GeneCards. MHC, major histocompatibility complex; SNP, single nucleotide polymorphism; *, SNP tags HLA-B57*01 in Europeans; **, SNP tags HLA-B57*03 in Africans; NR, no records.

The first HIV GWAS report unraveled genome-wide contributions of host genetic markers in the control of HIV infection in Europeans and Caucasians (Fellay et al., 2007). Consistent with previous candidate gene studies, in the Fellay et al. (2007) study, the major genetic determinants of HIV set point were in *HLA B* and *C* loci. These findings were replicated in subsequent GWAS of HIV-1 in European/Caucasian populations, with additional loci being identified (Dalmasso et al., 2008; Fellay et al., 2009; Le Clerc et al., 2009; Limou et al., 2009; Herbeck et al., 2010; Pereyra et al., 2010; Le Clerc et al., 2011). In most studies, the HIV-1 phenotypes of interest were viral set point and progression to disease (Fellay et al., 2007; Dalmasso et al., 2008; Fellay et al., 2009; Le Clerc et al., 2009; Limou et al., 2009; Herbeck et al., 2010; Pereyra et al., 2010; Le Clerc et al., 2011; Troyer et al., 2011; van Manen et al., 2011; Bartha et al., 2013).

The effects of the following SNPs were consistently replicated in European samples: *HCP5* rs2395029, *HLA-C rs9264942, ZNRD1 rs9261174, NOTCH4 rs8192591, and C6orf48 rs9368699*. The most replicated SNP was *HCP5* rs2395029 (which tags HLA-B*5701). The minor allele G of *HCP5* rs2395029 led to a reduction in viral load and delayed progression (Fellay et al., 2007; Dalmasso et al., 2008; Fellay et al., 2009; Limou et al., 2009; Pereyra et al., 2010; Le Clerc et al., 2011; Bartha et al., 2013). All these SNPs are located within the HLA region in chromosome 6. The HLA region is attributed by a long pattern of LD which makes it challenging to pinpoint the causal variant within this region. It has been observed that the minor allele of *HCP5* rs2395029 is often observed with the controlling C allele *HLA-C*rs9264942, both leading to a protective effect (Fellay et al., 2007; Fellay et al., 2009). At gene level, besides *HCP5*, *HLA-B*, and *HLA-C*, the following were also overrepresented: *TNXB*, *ZNRD1*, *RNF39*, and *MICB*. The role of these genes was delayed progression [*ZNRD1*, *RNF39* (Fellay et al., 2007; Fellay et al., 2009; Limou et al., 2009)] and reduction in viral load [*TNXB* and *MICB* (Dalmasso et al., 2008; Limou et al., 2009; Herbeck et al., 2010)]. Collectively, discovered SNPs within the European populations explained not more than 20% of the variability in viral load. More details on the identified SNPs can be found in the original publications and **Table 2**.

One of the major caveats of GWAS is the stringent threshold (p-value = 5.0 × 10^-8^) which accounts for multiple testing of about one million SNPs. As a result of this, SNPs with small effect sizes are not detected by GWAS and thousands of samples are required to effectively perform GWAS. In a study of progression in 404 Europeans, no SNP reached genome-wide significance (van Manen et al., 2011). The first GWAS of HIV-1 acquisition in European populations detected 11 SNPs which were genome-wide significant. Despite the large sample size, over 6,000 participants in each case/control category, these effects were disqualified due to frailty bias. Nonetheless, the study revealed a genome-wide association significance between imputed *CCR5*-Δ32 and HIV-1 acquisition [**Supplementary Table 1** (McLaren et al., 2013)]. The outcomes of these studies also show that the defining and selection of GWAS phenotypes should be handled with care (van Manen et al., 2011; McLaren et al., 2013).

GWAS of HIV-1 have also been performed in other populations, African Americans, Hispanics, Chinese, and Africans (Pelak et al., 2010; Pereyra et al., 2010; Petrovski et al., 2011; Wei et al., 2015; Xie et al., 2017). Pereyra et al. identified an intergenic SNP rs2523590 in Hispanics, Europeans, and Americans. The minor allele of this SNP was associated with low viral loads (Pereyra et al., 2010). Two GWAS of African Americans identified the SNP rs2523608 which tags the HLA-B*5703 allele in people of African descent. Having the minor allele of the SNP leads to a reduction in viral load (Pelak et al., 2010; Pereyra et al., 2010). When considered alone, HLA-B*5703 had the strongest signal and accounted for about 10% of the variation in viral load set point. Participants who had the HLA-B*5703 allele (absent in Europeans) showed improved viral control of the same magnitude with that observed in Europeans carrying the HLA-B*5701 allele (Pelak et al., 2010). The HLA-B*5701 allele was present in African Americans because of admixture at 0.3% and had little contribution to HIV-1 control. The implications of different alleles of HLA-B*57 in two populations of different ancestries emphasizes the importance of investigating rare variants that might affect HIV infection distinctly in different populations.

The first and only HIV-1 GWAS in the Chinese population was reported in 2015 (Wei et al., 2015). Several novel SNPs that correlated with HIV-1 viral load set point were identified, albeit not genome-wide significant. The top signals such as rs2442719 were observed in the HLA region. The authors highlighted that they observed no significant association for the highest peaks identified in Caucasians and African Americans (*HCP5* rs2395029 and *HLA-B* rs2523608 respectively), this indicating ethnic specificity of the variant associations [**Supplementary Table 1** (Wei et al., 2015)].

The *CCR5*-Δ32 variant that is associated with resistance to HIV infection in people of European ancestry is rare in African populations (Fowke et al., 1996; Joubert et al., 2010; Lingappa et al., 2011; Petrovski et al., 2011). Since *CCR5* variants were the only confirmed genetic markers that could influence HIV-1 acquisition in people of non-African ancestry, few studies were conducted to discover host genetics underlying HIV-1 acquisition in African populations (Kenya, Uganda and Tanzania, South Africa and Botswana (Lingappa et al., 2011), and Malawi (Petrovski et al., 2011). Lingappa et al. additionally investigated host genetics of viral control. These studies failed to detect signals of association between genetic variants and susceptibility to HIV-1 nor viral control. The authors postulated several arguments to explain the failure to detect signals: (1) susceptibility to HIV-1 might be due to non-genetic factors such as mode of transmission and viral sequence variability, (2) common or rare variants in an African populations might have not been represented in the chip used for genotyping, or (3) these weak signals may be due to the authors’ insufficient sample size (Lingappa et al., 2011; Petrovski et al., 2011).

The most recent HIV-1 GWAS was also conducted in an African (Botswana) population (Xie et al., 2017). This is the first GWAS to reveal significant variants associated with HIV-1 acquisition and progression in an African population. Moreover, two SNPs which had never been reported to associate with HIV infection nor progression were identified. These were SNPs rs2535307 and kgp22385164 located near *HCG22* (immune regulatory gene within chromosome 6) and *CCNG1* (encodes a cyclin that controls cell cycle; within chromosome 5) genes, respectively. The minor alleles of both SNPs led to lower *CD4* T-cell counts and high viral loads. *HCG22* was associated with progression and acquisition of HIV1-C, while *CCNG1* was associated with progression only in the Botswana cohort [**Table 2** (Xie et al., 2017)]. A validation test was done in data from three independent American cohorts, and no significant association was observed around the two genes (*HCG22*and *CCNG1*). SNPs that were found to associate with HIV infection and progression in previous studies did not appear in the top 100,000 p-values in this study (Xie et al., 2017).

**Figure 4:**
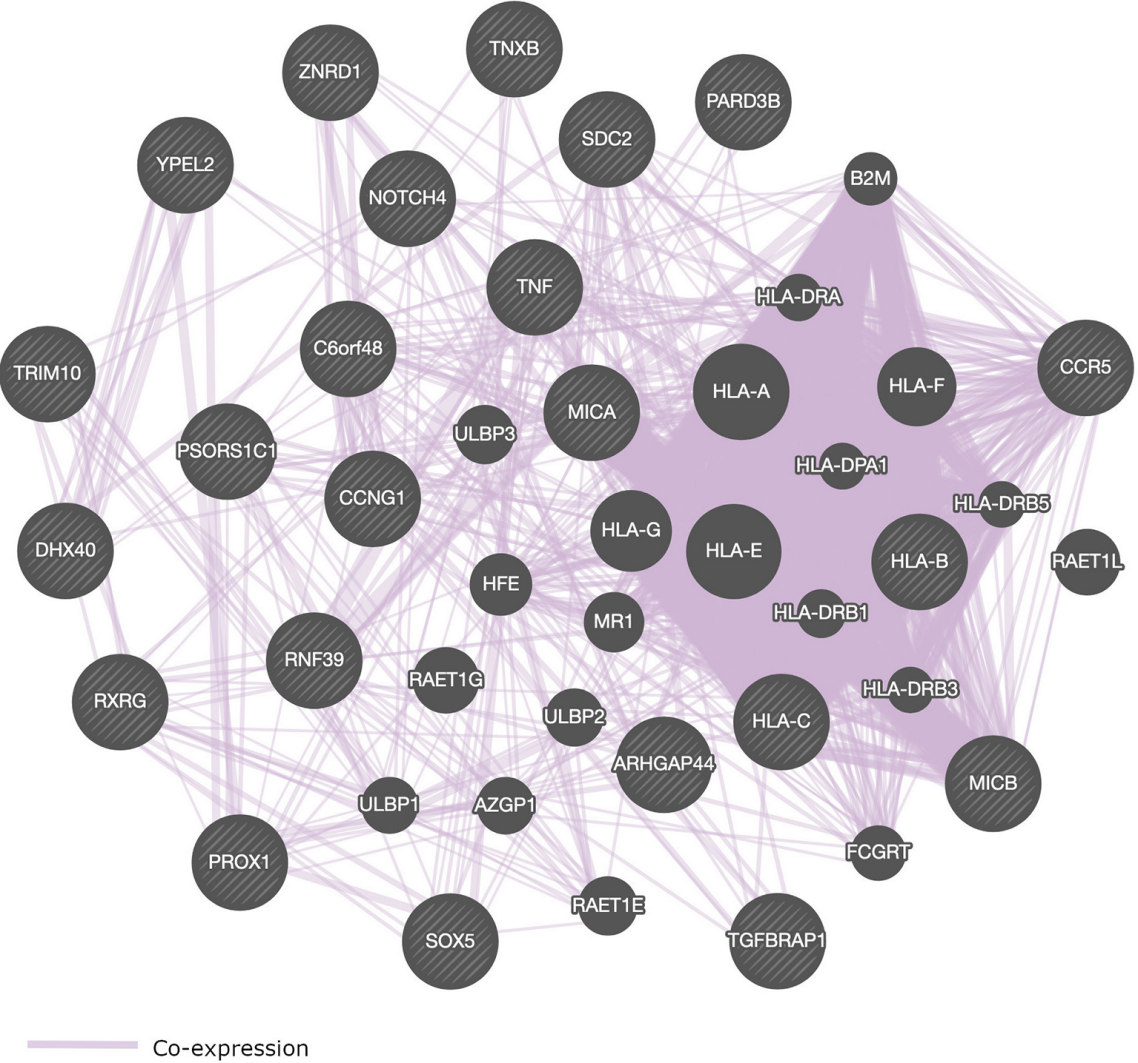
Pathway interaction network of genome-wide significant genes that control HIV-1 phenotypes. SNPs within or close to these genes are associated with the control of HIV-1. Striped circles indicate genes which have been identified from GWAS of HIV-1. Circles without stripes indicate genes putatively involved in the same pathway with genes implicated in HIV-1 phenotypes *via* GWAS. Purple lines represent gene co-expressions. The following genes could not be recognized by GeneMANIA software: *HCP5*, *PSORS1C3*, *AL671883.2*, *DHFRP2*, and *HCG22*.

Most of the SNPs that were identified in GWAS of HIV-1 are located within genes that play a role in immune response (**Table 2** and **Figure 4**). We present in **Figure 4**, a pathway interaction network of host candidate genes from GWAS of HIV-1 (**Table 2**) using GeneMANIA software (Warde-Farley et al., 2010). Out of the 28 genes in **Table 2**, GeneMANIA was able to predict the functions of 23 genes. Other genes that GeneMANIA deemed biologically and/or transcriptionally similar to the HIV-1 candidate genes from GWAS are also added to the network. According to databases accessed by GeneMANIA, the interactive genes are co-expressed with identified genes from GWAS of HIV-1 (**Table 2**). This suggests that more robust GWAS methods may pinpoint novel HIV-associated variants within genes from this broad gene-based network.

## GWAS of HIV-1 in Southern Africa

Only three GWAS of adult African populations have been published to date (Lingappa et al., 2011; Petrovski et al., 2011; Xie et al., 2017). These studies investigated common variants in Southern African populations of Malawi, Botswana, and South Africa. No variant reached genome-wide significance in two of these studies (Lingappa et al., 2011; Petrovski et al., 2011), while only two SNPs that had never been associated with HIV-1 acquisition and progression before were reported in the Botswana cohort (Xie et al., 2017). This lack of significant GWAS results is likely due to (1) lack of power from both sample size and statistical approaches used in both studies, (2) that the variants on microarrays could not tag the causal variants due to different LD patterns and different haplotype block structures between populations, or (3) that populations of Southern Africa use different mechanisms to control HIV-1C as a result of genetic diversity compared to previously studied populations.

## Conclusions and Perspectives

Southern Africa has a unique genetic architecture with evident structure between the populations within the region. The haplotype data of Khoe-San and Southern Bantu ethnolinguistic groups are currently absent from existing haplotype reference panels such as 1000 Genomes Project. Studies of population genetics and ancient DNA in Southern Africa are likely to provide new opportunities to discover novel disease susceptibility loci and refine gene-disease association signals. Southern Africans harbor ancient genetic diversity, as well as historical admixture, which can lead to complexities in (a) the design of studies assessing the genetic determinants of diseases and human variation, and (b) approaches for reconstructing DNA segments (sequence alignment) and variant discovery.

This review provided a broad discussion of genetic diversity in Southern Africa and its implication in the genetic architecture of HIV-1 phenotypes. In addition, we posited the advances made in HIV-1 GWAS. Few GWAS of HIV have been conducted using adult populations from Southern Africa. This is not enough considering that the region has the highest social and economic burden of HIV. Despite the insufficient sample sizes, HIV-1 GWA studies have reaffirmed the role of HLA in controlling HIV infection. *HLA-B*, *HLA-C*, *ZNRD1*, *RFN39*, and *HCP5* genes continue to be the most influential players in the outcome of HIV infection. However, a lot of implicated genes in GWAS reached significance by virtue of LD between the putative genes and the discovered variants. It is worthy to note that current findings from HIV-1 GWAS have not yet had a major impact on therapeutic optimization.

Critically, there has been a considerable amount of failure to replicate HIV-1 GWAS results across studies with some of the reasons being small sample sizes, different LD patterns between populations (causal SNPs probably not in the same haplotype block as tag-SNPs), and different criteria of phenotypic stratification. Nonetheless, many studies revealed novel variants which were not reported in previous studies. Two novel SNPs associated with HIV progression and acquisition were discovered in a Southern African population. This might imply that different networks of genes control HIV-1 in different regions. Given this, we hypothesize that functional categories of the genome may contribute disproportionately to the predisposition and resistance to HIV-1.

High genetic diversity, multi-wave genetic mixture, and low LD in the case of Southern African populations constitute a challenge in identifying genetic variants with modest risk or protective effect against HIV-1. Current GWAS of HIV-1 in Southern Africa were performed mostly in homogeneous populations; Malawi GWAS had a total of 1,532 Chichewa-speaking individuals, while the Botswana GWAS had a total of 556 individuals of undisclosed ethnicity. The Lingappa et al. study had 798 in total including 191participants of undisclosed ethnicity from Southern Africa. These sample sizes are not well powered to detect genome-wide significant SNPs of intermediate and small effect. Furthermore, considering the high genetic diversity and diverse patterns of admixture within Southern Africa, the populations used in the two GWAS of HIV-1 do not represent the diversity in the region. Thus, the findings of these studies cannot be effectively applied in other populations within the region. Hence, we need an inclusion of other populations in GWAS of HIV-1 in Southern Africa. More studies of admixture mapping should also be done to investigate the genetic role of different ancestries in HIV-1 phenotypes.

Moreover, factors such as host microbiome, viral genomics, epigenetics, social factors, and environmental factors can further contribute to HIV-1 acquisition, transmission, and progression. However, these components are not accounted for in current developed GWAS approaches that are mostly tailored for populations with long range of LD and haplotypes of European populations. These current GWAS approaches may limit the power of detecting possible associated variants and the ability to predict HIV-1 predisposition/resistance in the African context, in particular, Southern Africa. Therefore, failure to leverage these characteristics in modeling their joint contributions will be an obstacle to fully understand HIV-1 phenotype variability for an efficient and effective personalized therapy.

To date, GWAS of HIV-1 used Eurocentric microarrays for genotyping. Like other complex traits, HIV-1 phenotypes are polygenic; these traits are influenced by many rare variants of small effect sizes that microarray-based GWAS may fail to detect. However, other unbiased methods such as whole-genome or whole-exome sequences have the power to identify not only rare genetic variants but can also uncover more novel variants. Additionally, GWAS of Southern Africa may use SNP arrays that have a better coverage and representation of African genotypes and LD structures such as the recently developed Human Heredity and Health in Africa Consortium (H3Africa) SNP array. Other cost-effective approaches would be to develop or optimize targeted sequencing approaches such as the recently developed single primer enrichment technology. The advantage of target enrichment approaches is that they offer scalability and avoid the ascertainment bias that is common with microarray genotyping.

Exploring information from GWAS summary statistics provides a new paradigm to GWAS and might enable a complete characterization of genetic susceptibility/resistance to a disease. To account for the missing heritability of HIV-1, we suggest to further (a) examine whole genome or whole exome sequences of Southern African populations to uncover population-specific variants, (b) investigate functional categories of the genome and cell type-specific elements to estimate their enrichment and polygenic contribution to heritability of HIV-1, (c) perform a meta-analysis of previous HIV-1 GWAS results of African populations, (d) predict HIV-1 polygenic risk score, and (e) use robust methods for aggregation of GWAS signals to augment the identification of implicated genes and pathways or sub-networks. Importantly, it is worthy to note that HIV-1 polygenic risk scores based on European GWAS results may likely be poor predictors in Southern African populations because of differences in haplotype structure, patterns of LD (i.e., LD-tagging), and population-specific variation.

Changes in the host gene expression (or regulation) are a crucial stage in biological mechanisms underlying resistance and predisposition to HIV-1, and yet our current understanding of gene regulation is still limited. Without deep understanding of host gene regulation and the paucity in available tools for examining regulation, the transition from GWAS results to biological insights (biological mechanisms, genes involved, and the direction of causality) will remain a challenge.

Studies of different populations (including sub-Saharan Africa) have revealed that women with diverse vaginal microbiota have an increased risk of acquiring and transmitting HIV. However, these findings were not correlated with host genetics. Most studies investigating HIV and the gut microbiome were focused in non-African populations. It is known that the gut microbiome of populations differ geographically; therefore, it will be challenging to translate data from developed countries to populations in developing countries. Given the influences of (1) host genetics on the microbiome (gut, oral, genital, rectal, and lung), (2) environmental factors on microbiome profile, (3) HIV-1 infection on the microbiome, and (4) microbiome on host phenotypes, it is apparent that integrating the microbiome in host GWAS will reveal important insights and launch the first steps towards establishing causality in HIV-1 GWAS. The integrative analysis will be critical to comprehend the role and mechanisms of host genetics, the microbiome, and environment in the manifestation of HIV-1 infections.

## Author Contributions

PT and EC conceived, structured, and wrote the content of the manuscript.

## Funding

This work was supported through the sub-Saharan African Network for TB/HIV Research Excellence (SANTHE), a DELTAS Africa Initiative [grant # DEL-15-006]. The DELTAS Africa Initiative is an independent funding scheme of the African Academy of Sciences (AAS)’s Alliance for Accelerating Excellence in Science in Africa (AESA) and supported by the New Partnership for Africa’s Development Planning and Coordinating Agency (NEPAD Agency) with funding from the Wellcome Trust [grant # 107752/Z/15/Z] and the UK government. The views expressed in this publication are those of the authors and not necessarily those of AAS, NEPAD Agency, Wellcome Trust, or the UK government. The authors would also like to thank the National Research Foundation of South Africa for funding (NRF) [grant # RA171111285157/119056].

## Conflict of Interest

The authors declare that the research was conducted in the absence of any commercial or financial relationships that could be construed as a potential conflict of interest.
