# Supplementary material for: Population Structure and Implications on the Genetic Architecture of HIV-1 Phenotypes Within Southern Africa
Source: Front Genet. 2019 Sep 27;10:905. doi: 10.3389/fgene.2019.00905 (PMC6777512; doi:10.3389/fgene.2019.00905)
Supplement: Supplementary file 1 [file Table_1.docx]

Supplementary Material

**Supplementary table 1: Genetic polymorphisms significantly associated with HIV-1 acquisition, viral load set point and progression in GWAS**

Chr: chromosome, -: information not available, VL: viral load, OR: odds ratio, RH: relative hazard, OR^a^: odds ratio given for the minor allele where OR > 1 is means a protective effect.

| SNP | Chr | Gene | HIV-1 phenotype | Effect | Effect size | (P-value) | Population (n) | References | |
| --- | --- | --- | --- | --- | --- | --- | --- | --- | --- |
| rs2395029 | 6 | *HCP5* | VL set-point | Low VL | β = -1.0 | (9.36E-12) | Europeans  Caucasians  (486) | (J. Fellay et al., 2007) |  |
| rs9264942 | 6 | *HLA-C* | VL set-point | Low VL | β = -0.39 | (3.77E-9) |  |  | |
| rs9261174 | 6 | *ZNRD1* | VL set point | Low VL |  | (7.11E-3) |  |  | |
| rs9261174 | 6 | *ZNRD1* | Progression | Delayed progression |  | (3.89E-7) |  |  | |
| rs2395029 | 6 | *HCP5* | Progression Reservoir | Low VL  Low reservoir | β = -0.540 | (672E-7) | Europeans  (605) | (Dalmasso et al., 2008) | |
| rs13199524 | 6 | *TNXB* | Progression Reservoir | Low VL | β = 0.255 | (5.70E-5) |  |  | |
| rs3093662 | 6 | *TNF* | Progression Reservoir | Low VL reservoir | β = -0.247 | 2.18E-5 |  |  | |
| rs12198173 | 6 | *TNXB* | Progression Reservoir | Low VL Low reservoir | β = 0.240 | 1.28E-4 |  |  | |
| rs6503919 | 17 | *DDX40 YPEL2* | Reservoir | Low reservoir | β = -0.211 | 2.00E-6 |  |  | |
| rs2575735 | 8 | *SDC2* | Reservoir | Low reservoir | β = -0.176 | 1.34E-6 |  |  | |
| rs2395029 | 6 | *HCP5* | VL set-point | Low VL | - | 4.48E-35 | Caucasian  (2554) | (Jacques Fellay et al., 2009) | |
| rs9264942 | 6 | *HLA-C* | VL set-point | Low VL | - | 5.85E-32 |  |  | |
| rs259919 | 6 | *C6orf12* | VL set-point | - | - | 5.3E-4 |  |  | |
| rs9468692 | 6 | *TRIM10* | VL set-point | Low VL | - | 7.6E-4 |  |  | |
| rs9266409 | 6 | *HLA-B* | VL set-point | Low VL | - | 4.86E-14 |  |  | |
| rs8192591 | 6 | *NOTCH4* | VL set-point | Low VL | - | 9.02E-9 |  |  | |
| rs2395029 | 6 | *HCP5* | Progression | Delayed progression | - | 1.20E-11 |  |  | |
| rs9264942 | 6 | *HLA-C* | Progression | Delayed progression | - | 6.40E-12 |  |  | |
| rs9261174 | 6 | *ZNRD1* | Progression | Delayed progression | - |  |  |  | |
| rs3869068 | 6 | *ZNRD1* | Progression | Delayed progression | - | 1.80E-8 |  |  | |
| rs2074480 | 6 | *RNF39* | Progression | Delayed progression | - | 1.80E-8 |  |  | |
| rs7758512 |  | *ZNRD1* | Progression | Delayed progression | - | 1.80E-8 |  |  | |
| rs9261129 | 6 | *HCG8, ZNRD1* | Progression | Delayed progression | - | 1.80E-8 |  |  | |
| rs2301753 | 6 | *RNF39* | Progression | Delayed progression | - | 1.80E-8 |  |  | |
| rs2074479 | 6 | *RNF39* | Progression | Delayed progression | - | 1.80E-8 |  |  | |
| rs2395029 | 6 | *HCP5* | Set-point | Low VL | OR = 3.47 | 6.79E-10 | Europeans (275 nonprogressors, 1352 controls) | (Limou et al., 2009) | |
| rs1245371 | 6 | *RNF39* | Progression | Delayed progression | - | 9.21E-7 |  |  | |
| rs9368699 | 6 | *C6orf48* | Set-point | Low VL | - | 1.84E-11 |  |  | |
| rs3823418 | 6 | *PSORS1C1* | Set-point | Low VL | - | 1.4E-8 |  |  | |
| rs2248462 | 6 | *MICB* | Set-point | Low VL | - | 4.26E-8 |  |  | |
| rs2516509 | 6 | *MICB* | Set-point | Low VL | - | 4.95E-8 |  |  | |
| rs10484554 | 6 | *HLA-C* | Set-point | Low VL | - | 6.27E-8 |  |  | |
| rs3815087 | 6 | *PSORS1C1* | Set-point | Low VL | - | 1.46E-7 |  |  | |
| rs259940 | 6 | *ZNRD1* | Progression | Delayed progression | - | 2.04E-6 |  |  | |
| rs4118325 | 1 | *Intergenic* | Progression | Delayed progression | OR = 0.24 | 6.09E-7 | Europeans (1352 controls; 85 rapid progressors) | (Le Clerc et al., 2009) | |
| rs1522232 | 12 | *SOX5* | Progression | Delayed progression | OR = 0.45 | 1.80E-6 |  |  | |
| rs1360517 | 9 | *Intergenic* | Progression | Rapid progression | OR = 3.09 | 3.27E-6 |  |  | |
| rs3108919 | 8 | *Intergenic* | Progression | Rapid progression | OR = 2.13 | 3.86E-6 |  |  | |
| rs10800098 | 1 | *RXRG* | Progression | Rapid progression | OR = 3.29 | 3.86E-6 |  |  | |
| rs10494056 | 1 | *Intergenic* | Progression | Delayed progression | OR = 0.27 | 4.29E-6 |  |  | |
| rs12351740 | 9 | *Intergenic* | Progression | Rapid progression | OR = 3.46 | 6.63E-6 |  |  | |
| rs1020064 | 2 | *TGFBRAP1* | Progression | Delayed progression | OR = 0.34 | 7.04E-6 |  |  | |
| Haplotype of rs17762192, rs17762150 and rs1367951 | 1 | *PROX1* | Progression | Delayed progression | RH = 0.69 | 6.23E-7 | European Americans  (156) | (Herbeck et al., 2010) | |
| rs16899646 | 6 | *HLA-B* | Progression | Delayed progression | - | 1.33E-5 |  |  | |
| rs2248462 | 6 | *Intergenic* | Set-point | Low VL | - | 1.16E-2 |  |  | |
| rs2516422 | 6 | *MICB* | Set-point | Low VL | - | 5.12E-4 |  |  | |
| rs2395034 | 6 | *MICB* | Set-point | Low VL | - | 5.13E-4 |  |  | |
| HLA-B*5703 | 6 | *HLA-B* | Set-point | Low VL | - | 5.60E-10 | African Americans  (515) | (Pelak et al., 2010) | |
| rs2523608 | 6 | *HLA-B* | Set-point | Low VL | - | 2.29E-6 |  |  | |
| rs9264942 | 6 | *HLA-C* | VL set-point | Low VL | OR^a^ = 2.9 | 2.8E-35 | Europeans  (1712) | (Pereyra et al., 2010) | |
| rs2395029 | 6 | *HCP5* | VL set-point | Low VL | OR^a^ = 5.3 | 9.7E-26 |  |  | |
| rs4418214 | 6 | *MICA* | VL set-point | Low VL | OR^a^ = 4.4 | 1.4E-34 |  |  | |
| rs3131018 | 6 | *PSORS1C3* | VL set-point | Low VL | OR^a^ = 1.5 | 4.2E-16 |  |  | |
| rs2523608 | 6 | *HLA-B*5703* | VL set-point | Low VL | OR^a^ = 2.6 | 8.9E-20 | African Americans  (1233) |  | |
| rs2255221 | 6 | *HCP5* | VL set-point | Decreased VL | OR^a^ = 2.7 | 3.5E-14 |  |  | |
| rs2523590 | 6 | *DHFRP2* | VL set-point | Low VL | OR^a^ = 2.3 | 1.7E-13 |  |  | |
| rs9262632 | 6 | *HCG22* | VL set-point | Low VL | OR^a^ = 3.1 | 1.0E-8 |  |  | |
| rs2523590 | 6 | *DHFRP2* | VL set-point | Low VL | OR^a^ = 2.5 | 8.3E-8 | Hispanics  (667) |  | |
| rs2395029 | 6 | *HCP5* | Progression | Delayed progression | OR^a^ = 3.14 | 8.54E-15 | Europeans and Caucasians (n variable in cases, not > 270) | (Le Clerc et al., 2011) | |
| rs9368699 | 6 | *C6orf48* | Progression | Delayed progression | OR^a^ = 2.9 | 3.03E-10 |  |  | |
| rs8192591 | 6 | *NOTCH4* | Progression | Delayed progression | OR^a^ = 2.32 | 9.08E-27 |  |  | |
| rs2072255 | 17 | *RICH2* | Progression | Rapid progression | OR = 0.43 | 3.30E-26 |  |  | |
| rs11884476 | 2 | *PARD3B* | Progression | Delayed progression | RH = 0.3 | 3. 37E-9 | European Americans  (755) | (Troyer et al., 2011) | |
| CCR5-Δ32 | 3 | *CCR5* | Acquisition | Resistance to HIV-1 | OR = 0.2 | 5.0E-9 | (2,173) | (McLaren et al., 2013) | |
| rs2535307 | 6 | *HCG22* | Progression & acquisition | Rapid progression and Increased susceptibility | - | 3.72E-7 | Southern Africans (556) | (Xie et al., 2017) | |
| kgp22385164 | 5 | *CCNG1* | Progression | Rapid progression | - | 1.88E-6 |  | (Xie et al., 2017) | |
